# 

**Published:** 1885-11

**Authors:** 


### BOOKS AND PAMPHLETS RECEIVED.

---

MISCELLANEOUS REPRINTS. By James Craig, M. D. Jersey City, N. J.

INSOMNIA AND OTHER DISORDERS OF SLEEP. By Henry M. Lyman, A. M., M. D. W. T. Kenan, Chicago, Publisher. 1885.

INEBRIISM. By T. L. Wright, M. D. William G. Hubbard, Columbus, O., Publisher. 1885.

IS CRANIOTOMY UPON THE LIVING FŒTUS EVER JUSTIFIABLE? By Robert B. Dixon, M. D.

MEDICAL COMMUNICATIONS of the Massachusetts Medical Society. Vol. XIII, No. IV. 1885.

VAGINAL HYSTERECTOMY FOR CANCER. By A. Reeves Jackson, A. M., M. D.

THE MANAGEMENT OF LABOR. By Henry G. Landis, A. M., M. D. Lea Brothers & Co. Philadelphia. 1885.

A TREATISE ON NERVOUS DISEASES. By Samuel G. Webber, M. D. D. Appleton & Co. New York: 1885.

ABNORMAL POSITIONS OF THE HEAD—WHAT DO THEY INDICATE? By Edward Borck, A. M., M. D.

MEDICAL EDUCATION. By Henry Leffman, M. D. Reprint Proceedings Philadelphia Co. Medical Society.

TRANSACTIONS of the Medical Association of Alabama. 1885. Thomas A. Means, M. D., Secretary. Montgomery, Ala.

THE FIRST THREE YEARS OF CHILDHOOD. Chicago: A. N. Marquis & Co. 1885.

ADDRESS OF THE STATE BOARD OF HEALTH AND VITAL STATISTICS of the Commonwealth of Pennsylvania.

A NEW BANDAGE FOR FIXATION OF THE HUMERUS AND SHOULDER-GIRDLE. By Charles W. Dulles, M. D.

PRURITIC RHINITIS, or Hay Fever. By Thomas F. Rumbold, M. D. St. Louis. Medical Journal Publishing Co. 1885.

ELEMENTS OF MODERN MEDICINE. By R. French Stone, M. D., Indianapolis, Ind. New York: D. Appleton & Co. 1885.

MILK ANALYSIS AND INFANT FEEDING. By Arthur V. Meigs, M. D. Philadelphia: P. Blakiston, Son & Co. 1885.

THE THERAPEUTICS OF HIGH TEMPERATURES IN YOUNG CHILDREN. By William Perry Watson, A. M., M. D., Jersey City, N. J.

Fownes' Manual of Chemistry. Philadelphia: Lea Bros. & Co. 1885.

A Text-Book of Pharmacology, Therapeutics and Materia Medica. By T. Lauder Brunton, M. D., D. S. C., F. R. S. Philadelphia: Lea Bros. & Co. 1885.

A System of Obstetric Medicine and Surgery. By Robert Barnes, M. D., and Fancourt Barnes, M. D. Philadelphia: Lea Bros. & Co. 1885.

A System of Practical Medicine. By American Authors. Edited by William Pepper, M. D., LL. D., assisted by Louis Slare, M. D. Vol. III. Philadelphia: Lea Bros. & Co. 1885.

A Practical Treatise on Diseases of Children. By Alfred Vogel, M. D.. Translated and edited by H. Raphael, M. D. D. Appleton & Co. New York. 1885.

Typhoid or Enteric Fever—(Abortive Treatment.) By Edwin R. Maxson, M. D., A. M., LL. D., Syracuse, New York.

Epitome of Diseases of the Skin. By Louis A. Duhring, M. D. Reported by Henry Wile, M. D., Atlanta, Ga. Philadelphia: J. B. Lippincott Co. 1885.

The Respiratory Function of the Human Larynx. From Experimental Studies in the Physiological Laboratory of Harvard University. By Franklin H. Hooper, M. D. Boston

Tracts on Massage, No. 2. The Physiological Effects of Massage. Translated from the German of Reibneayer. By Benj. Lee, A. M., M. D. Philadelphia. 1885.

On Renal and Urinary Affections. By W. Howship Dickinson, M. D. Cantab., F. R. C. P. New York: Wm. Wood & Co. 1885.

Poisons, Their Effects and Detection. Vol. II. By Alex. Wynter Blyth, M. R. C. S. F. C. S. & C. New York: Wm. Wood & Co. 1885.

Transactions of the Texas State Medical Association, Seventeenth Annual Session. Austin: W. J. Burt, M. D., Secretary, F. E. Daniel, M. D., Chairman Publishing Committee. 1885.

